# Supplementary material for: Evaluating the Role of Corrals and Insects in the Transmission of Porcine Cysticercosis: A Cohort Study
Source: Pathogens. 2023 Apr 14;12(4):597. doi: 10.3390/pathogens12040597 (PMC10143142; doi:10.3390/pathogens12040597)
Supplement: Supplementary file 1 [file pathogens-12-00597-s001.zip › pathogens-2317962-supplementary.pdf]

pathogens

Article

Evaluating the Role of Corrals and Insects in the Transmission of Porcine Cysticercosis: A Cohort Study

Eloy Gonzales-Gustavson [1,*], Ian W. Pray [2], Ricardo Gamboa [3], Claudio Muro [3], Percy Vilchez [3], Luis Gomez-Puerta [1], Ana Vargas-Calla [1], Gabrielle Bonnet [4], Francesco Pizzitutti [5], Hector H. Garcia [3,6], Armando E. Gonzalez [1] and Seth E. O'Neal [2,3,*] on behalf of the Cysticercosis Working Group in Peru

[1] Departamento de Salud Animal y Salud Pública, Universidad Nacional Mayor de San Marcos, Lima 15081, Peru
[2] School of Public Health, Oregon Health & Science University and Portland State University, Portland, OR 97239, USA
[3] Center of Global Health, Universidad Peruana Cayetano Heredia, Lima 15202, Peru
[4] Centre for the Mathematical Modeling of Infectious Diseases, London School of Hygiene and Tropical Medicine, London WC1H 9SH, UK
[5] Geography Institute, Universidad San Francisco de Quito, Quito 170157, Ecuador
[6] Cysticercosis Unit, National Institute of Neurological Sciences, Lima 15003, Peru
* Correspondence: egonzalesg@unmsm.edu.pe (E.G.-G.); oneals@ohsu.edu (S.E.O.)

Citation: Gonzales-Gustavson, E.; Pray, I.W.; Gamboa, R.; Muro, C.; Vilchez, P.; Gomez-Puerta, L.; Calla, A.V.; Bonnet, G.; Pizzitutti, F.; Garcia, H.H.; et al. Evaluating the Role of Corrals and Insects in the Transmission of Porcine Cysticercosis: A Cohort Study. *Pathogens* **2023**, *12*, 597. https://doi.org/10.3390/pathogens12040597

Academic Editor: Hans-Peter Fuehrer

Received: 16 March 2023
Revised: 5 April 2023
Accepted: 12 April 2023
Published: 14 April 2023

Copyright: © 2023 by the authors. Licensee MDPI, Basel, Switzerland. This article is an open access article distributed under the terms and conditions of the Creative Commons Attribution (CC BY) license (https://creativecommons.org/licenses/by/4.0/).

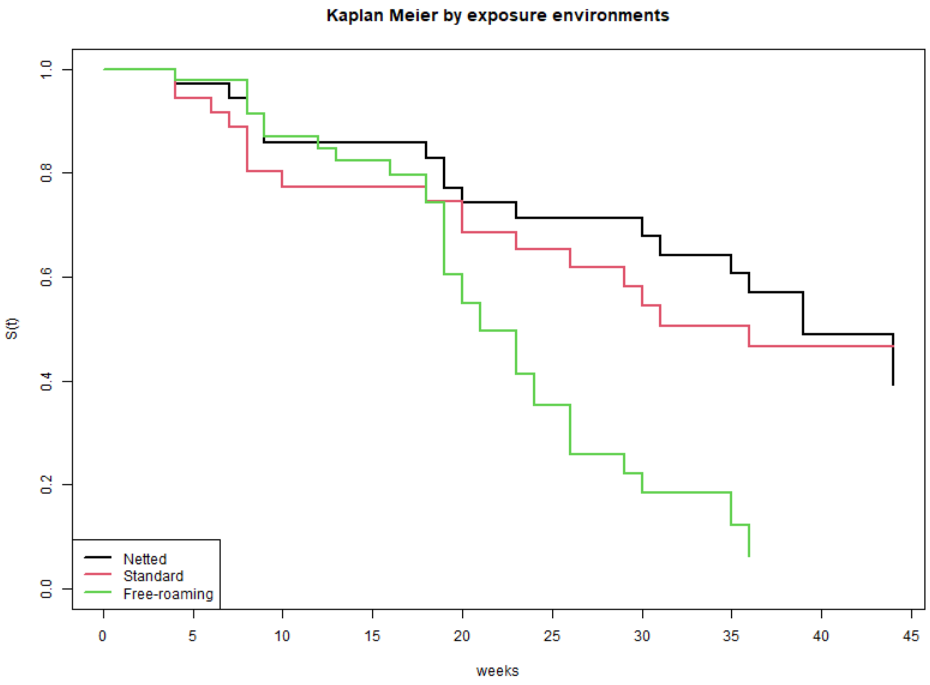

Figure S1. Kaplan Meier survival curve with the time to develop a EITB positive result between the three kinds of exposure environments evaluated: netted corrals, standard corrals, and free roaming.

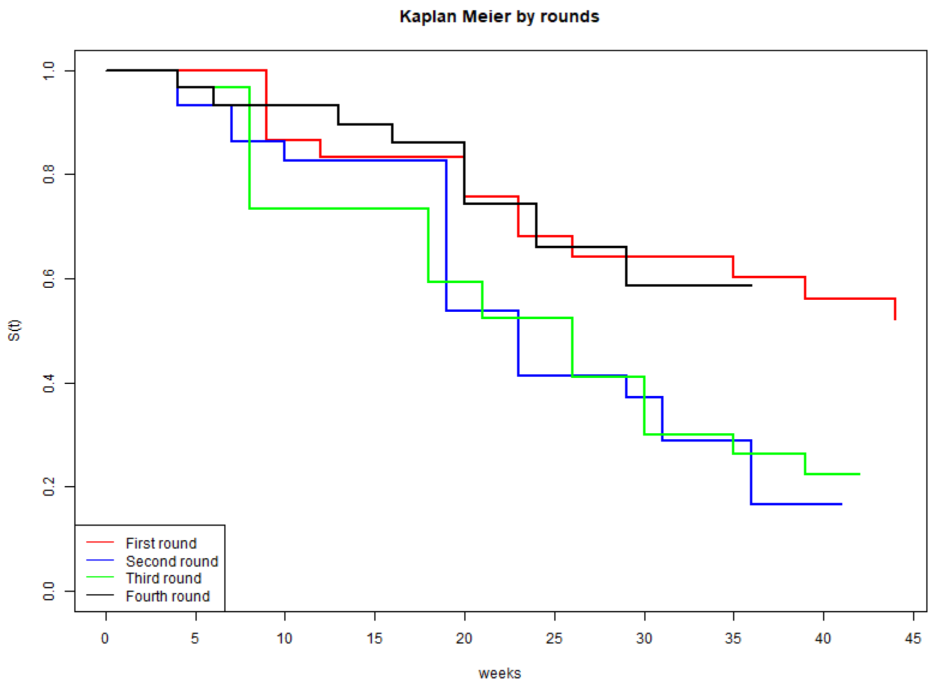

Figure S2. Kaplan Meier survival curve with the time to develop a EITB positive result between the four rounds evaluated.

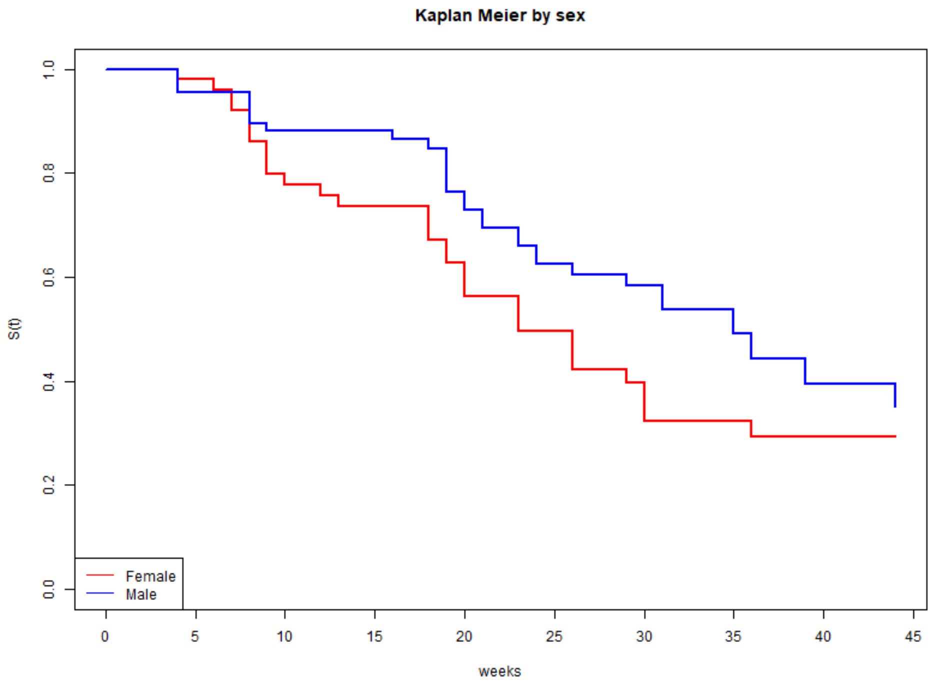

Figure S3. Kaplan Meier survival curve with the time to develop a EITB positive result between sex (male in blue and female in red) of pigs.

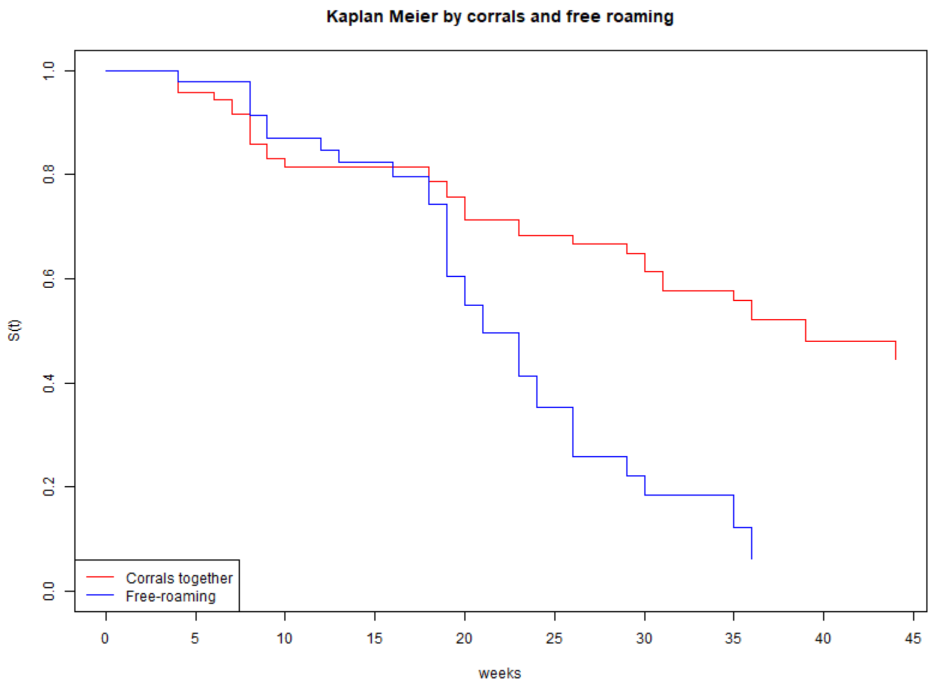

Figure S4. Kaplan Meier survival curve with the time to develop a EITB positive result between corrals grouped together (red) and free-roaming (blue).

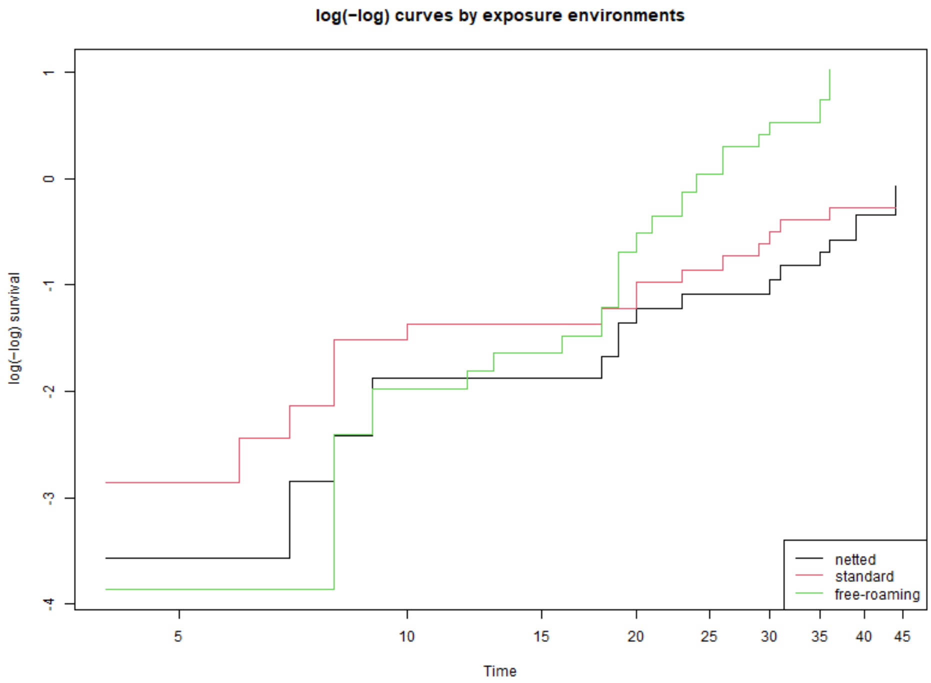

Figure S5. Estimated -ln(-ln) log(−log) survivor curves over the three categories of breeding system. Parallel curves are nor observed which assumes Proportional hazard is not satisfied.

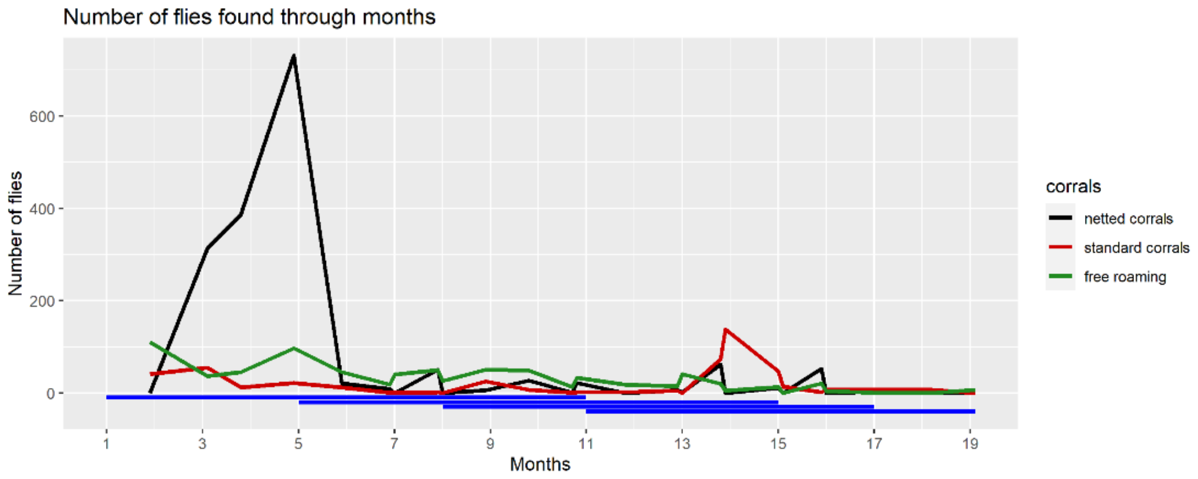

Figure S6. Number of flies caught through time (months) in the three breeding systems, blue lines at the bottom correspond to each round.

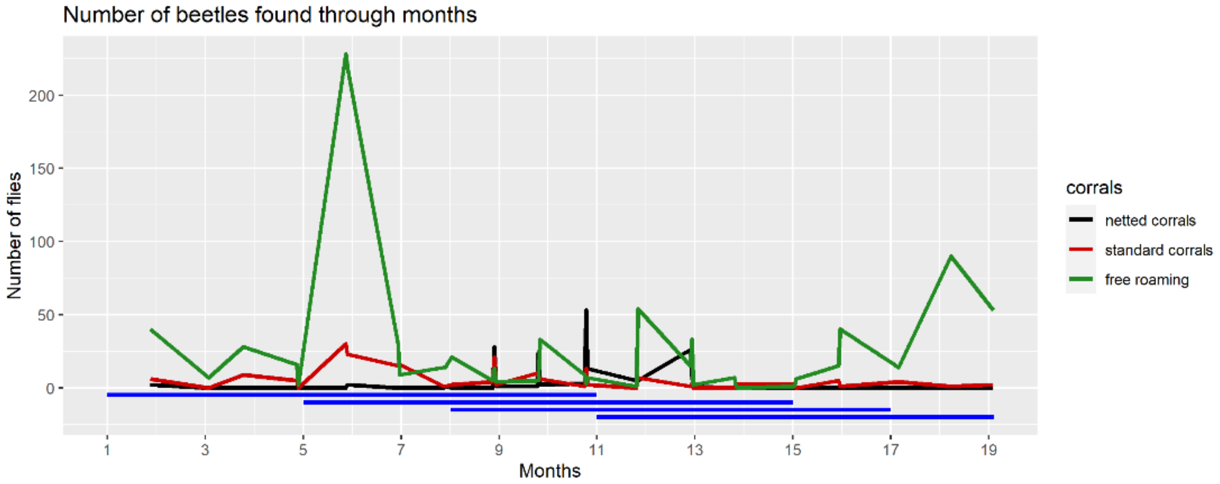

Figure S7. Number of beetles caught through time (months) in the three breeding systems, blue lines at the bottom correspond to each round.

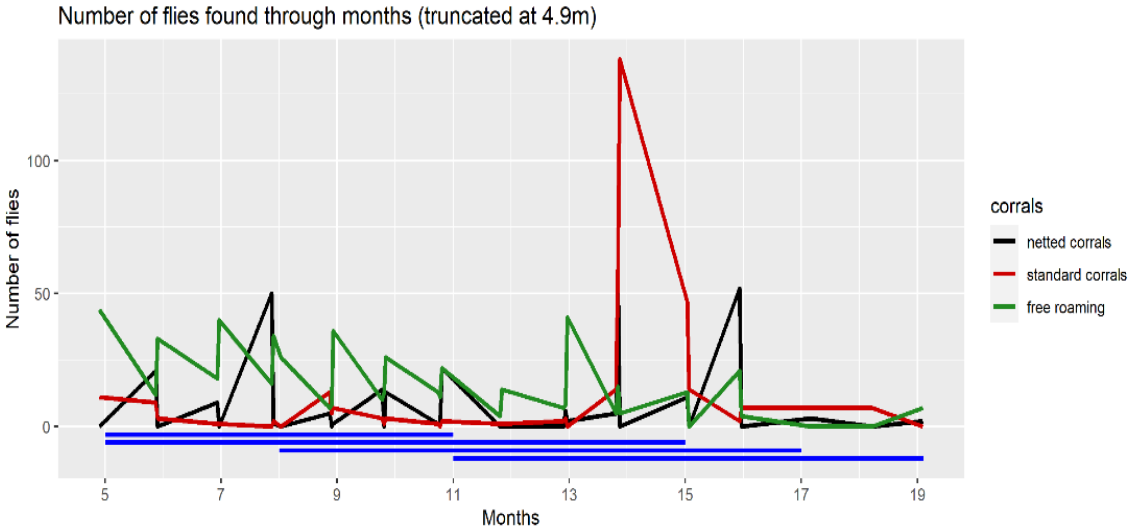

Figure S8. Number of flies caught through time (from 4.9 months into the study) in the three breeding systems, blue lines at the bottom correspond to each round.

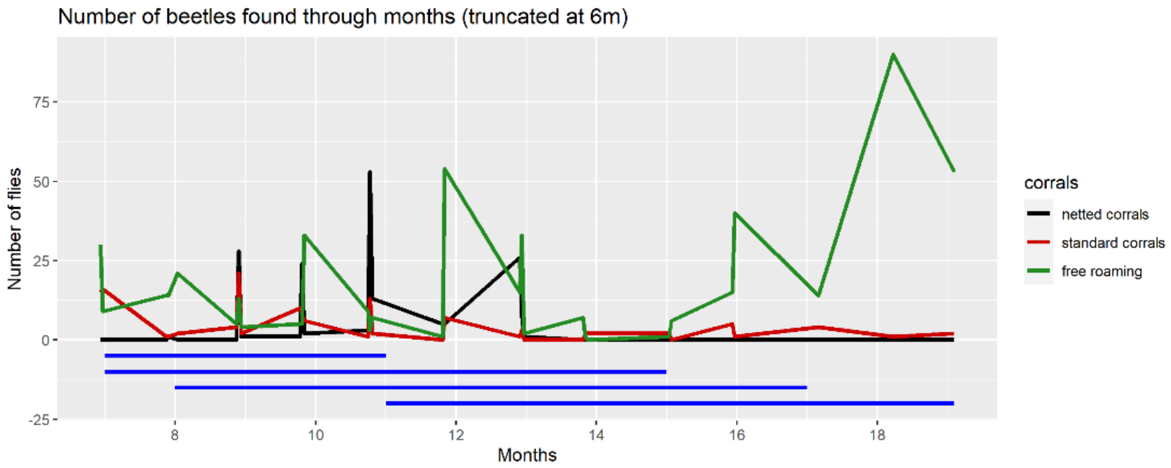

Figure S9. Number of flies caught through time (from 6 months into the study) in the three breeding systems, blue lines at the bottom correspond to each round.

Table S1. Cox proportional hazard model with three independent variables: breeding system, rounds and sex.

Variable	PH*	lower 0.95	upper 0.95	*p*-value
**Groups**				
Standard/Netted	1.30	0.66	2.58	0.4
Free roaming/Netted	3.18	1.70	5.97	0.0003
**Sex**				
Male/Female	0.62	0.38	1.01	0.056
**Round**				
Second / First	2.62	1.27	5.41	0.009
Third / First	2.40	1.18	4.91	0.016
Fourth / First	0.99	0.42	2.33	1.0

*PH: Proportional hazard.

Table S2. Goodness of fit for the model in Table S2.

Variable	chisq *	df	*p*-value
Exposure environment	12.96	2	0.002
Sex	1	1	0.3
Round	1.06	3	0.8
Global	14.57	6	0.02

* Chi-squared test.

Table S3. Goodness of fit for the model in Table 2.

Variable	chisq *	df	*p*-value
Round	2.03	3	0.57
Sex	1.03	1	0.31
Free roaming /Corrals (before 18 weeks)	1.41	1	0.24
Free roaming /Corrals (after 18 weeks)	0.27	1	0.6
Global	4.31	6	0.63

* Chi-squared test.

Table S4. Maximum number of reactive bands observed on the LLGP-EITB test throughout the observation period, among pigs in each exposure environment.

**Environment**	*T. solium* cysts at necropsy	number of maximum bands			
		0	1–2	3	4–7
Netted corral	no	19	15	1	0
	yes	0	0	0	0
Standard corral	no	20	13	2	1
	yes	0	0	0	0
Free roaming	no	7	11	4	0
	yes	2	5	5	3

Table S5. Number of pigs infected with *T. solium* by rounds (only in free roaming).

**Rounds**	**Negative**	**Positive**
First	8	0
Second	6	4
Third	3	7
Fourth	5	4

Pearson´s Chi-squared test, p-value = 0.027.

Table S6. Number of pigs infected with *T. solium* by sex (only in free roaming).

	**Negative**	**Positive**
**Female**	9	6
**Male**	13	9

Pearson´s Chi-squared test, p-value = 1.

Disclaimer/Publisher's Note: The statements, opinions and data contained in all publications are solely those of the individual author(s) and contributor(s) and not of MDPI and/or the editor(s). MDPI and/or the editor(s) disclaim responsibility for any injury to people or property resulting from any ideas, methods, instructions or products referred to in the content.
